# Nutritional behavior of cyclists during a 24-hour team relay race: a field study report

**DOI:** 10.1186/1550-2783-9-3

**Published:** 2012-02-06

**Authors:** Raúl Bescós, Ferran A Rodríguez, Xavier Iglesias, Beat Knechtle, Adolfo Benítez, Míchel Marina, Josep M Padullés, Priscila Torrado, Jairo Vazquez, Thomas Rosemann

**Affiliations:** 1 Institut Nacional d'Educació Física de Catalunya, Sports Sciences Research Group, Universitat de Barcelona, Spain; 2 Institute of General Practice and for Health Services Research, University of Zurich, Switzerland

**Keywords:** energy balance, ultra-endurance, macronutrient consumption, hydration, bicycling, weighed dietary record, descriptive study

## Abstract

**Background:**

Information about behavior of energy intake in ultra-endurance cyclists during a 24-hour team relay race is scarce. The nutritional strategy during such an event is an important factor which athletes should plan carefully before the race. The purpose of this study was to examine and compare the nutritional intake of ultra-endurance cyclists during a 24-hour team relay race with the current nutritional guidelines for endurance events. Additionally, we analyzed the relationship among the nutritional and performance variables.

**Methods:**

Using a observational design, nutritional intake of eight males (mean ± SD: 36.7 ± 4.7 years; 71.6 ± 4.9 kg; 174.6 ± 7.3 cm; BMI 23.5 ± 0.5 kg/m^2^) participating in a 24-hour team relay cycling race was assessed. All food and fluid intake by athletes were weighed and recorded. Additionally, distance and speed performed by each rider were also recorded. Furthermore, before to the race, all subjects carried out an incremental exercise test to determine two heart rate-VO_2 _regression equations which were used to estimate the energy expenditure.

**Results:**

The mean ingestion of macronutrients during the event was 943 ± 245 g (13.1 ± 4.0 g/kg) of carbohydrates, 174 ± 146 g (2.4 ± 1.9 g/kg) of proteins and 107 ± 56 g (1.5 ± 0.7 g/kg) of lipids, respectively. This amount of nutrients reported an average nutrient intake of 22.8 ± 8.9 MJ which were significantly lower compared with energy expenditure 42.9 ± 6.8 MJ (*P *= 0.012). Average fluid consumption corresponded to 10497 ± 2654 mL. Mean caffeine ingestion was 142 ± 76 mg. Additionally, there was no relationship between the main nutritional variables (*i.e*. energy intake, carbohydrates, proteins, fluids and caffeine ingestion) and the main performance variables (*i.e*. distance and speed).

**Conclusions:**

A 24-hour hours cycling competition in a team relay format elicited high energy demands which were not compensated by energy intake of the athletes despite that dietary consumption of macronutrients did not differ to the nutritional guidelines for longer events.

## Background

Ultra-endurance competitions are defined as endurance performances of more than six hours of duration [1]. Traditionally, ultra-endurance races are held as solo events in attempts to challenge the limits of human endurance. However, the increased popularity of these competitions in recent years has led to different formats of participation, such as team relays with four riders per team [2]. In comparison with solo events where athletes perform a continuous exercise (> 6 hours) at a mean intensity of ~60% of maximum oxygen uptake (VO_2_max) [3], team relay competitions elicit intermittent exercise at a mean intensity above 75% of VO_2_max [4,5].

The nutritional strategy during ultra-endurance events is an important factor that athletes should plan carefully before the race. The amount and the source of energy intake, fluid replacement, as well as the ingestion of stimulants such as caffeine are important factors directly linked to sport performance in endurance events [6,7]. In relation with the energy demands, several studies have assessed the nutritional requirements and behavior of cyclists during solo events [8-10]. However, there is a lack of information about the energy requirements of athletes competing in a team relay. To the best of our knowledge, only one study has estimated the energy expenditure and dietary intake of cyclists during one competition of 24-hour in a team relay format [4]. Surprisingly, this study showed that athletes ingested only 45% of their estimated energy expenditure during the race. These data are in concordance with results reported in solo riders [8-10] despite that in team relay events, cyclists have a considerable time to recover between the bouts of exercise [4,5].

There is broad evidence that during longer events the energy replacement should be mainly based on food rich in carbohydrate since glycogen stores in the body are limited [11]. This fact could be even more important in intermittent high-intensity competitions such as ultra-endurance team relay events where athletes are performing several bouts of exercise at higher intensity with limited recovery period between them. When carbohydrates are not available, or available only in a limited amount, the intensity of exercise must be reduced to a level where the energy requirement can be met by fat oxidation [7,12]. The most recent studies in laboratory conditions indicate that the adequate amount of carbohydrate intake to reduce muscle glycogen depletion and optimize carbohydrate oxidation during prolonged exercise is ~1.5 g/min [13-15].

Other important issues during ultra-endurance events are both fluid replacement and caffeine ingestion. For instance, it is known that the consumption of beverages containing electrolytes and carbohydrates in a concentration of 6 - 8% enhances performance compared to the consumption of plain water [16]. Consumption of caffeine has been also linked to an improved exercise tolerance [17]. Doses of between 1.5 and 3.5 mg/kg have been found to improve time-trial performances in laboratory studies [18]. The mechanisms to explain benefits of caffeine ingestion are based on an increased utilization of plasma free fatty acids and reduced oxidation of muscle glycogen [19], as well as favorable changes in the central nervous system [20]. However, there is a lack of data indicating the hydration pattern and caffeine consumption followed by cyclists during ultra-endurance team relay competitions.

Accordingly, the primary aims of this study were (1) to describe the dietary energy intake of ultra-endurance cyclists participating in a 24-hour team relay competition, (2) to compare it with the current recommendations for longer events [6,7] and (3) to analyze the correlation between the nutritional intake and the variables of race performance such as completed distance and reached mean speed. We hypothesized that dietary intakes of athletes competing in a 24-hour ultra-endurance cycling race differ to the current nutritional recommendations for longer events, thus, leading to a high energy deficit. Some factors such as appetite suppression and gastro-intestinal distress can reduce the dietary intake during longer competitions. In addition, these disturbances can affect the performance of athletes leading to a decrease in performance during the race. This information is needed to expand the limited knowledge of the nutritional behavior of athletes during these types of events, as well as to report new information which could be useful for nutrition professionals to design an adequate nutritional strategy for athletes.

## Methods

### Design of the study

An observational field study at the 24-hour cycle race of Barcelona (Spain) was used for this research. The competition started at 19:00 hrs and consisted of completing the maximum distance possible during the 24-hour period, on a closed road circuit of 3,790 meters in length, and 60 meters of altitude per lap. Within the circuit, all the athletes had a box where they ingested food and performed their relays. The time and average speed of each cyclist was recorded on completion of each lap. The strategy chosen by the athletes during the race was up to them where every team decided the order and duration of the effort. The average temperature during the whole event was ~27.5°C (range: 24.6 - 31.0) and relative humidity was at ~53.9% (range: 33.0 - 72.0). The mean velocity of wind was at ~1.7 m/s (range: 0.6 - 3.0).

### Subjects

A total of eight experienced, non-professional, male athletes (6 cyclists and 2 triathletes), voluntarily participated in this study (see Table 1). The athletes were contacted by the researchers via phone between two and three weeks before the race. This race was the first experience in an ultra-endurance team relay cycling event for all athletes. The subjects had 12.9 ± 8.8 years of experience in endurance events, and their average weekly training volume was from 15 hours up to a maximum of 30 hours, with a total volume between 800 and 1,000 hours per year. They were all members of the Spanish Cycling or Triathlon Federations and, up to the start of the study, reported no related medical illnesses. All the subjects passed a medical examination and gave their informed written consent, approved by the Ethics Committee of the Catalonian Sports Council, prior to their participation.

**Table 1:** Physical and physiological characteristics of the subjects

*Subjects*	1	2	3	4	5	6	7	8	M ± SD
Age (years)	34.4	39.7	29.6	38.3	43.3	39.8	31.0	37.5	**36.7 ± 4.7**
Height (cm)	167.0	172.4	189.1	165.1	177.6	173.5	176.0	176.0	**174.6 ± 7.3**
Body mass (kg)	65.3	68.9	79.9	65.7	73.9	74.5	72.5	72.4	**71.6 ± 4.9**
BMI (kg·m^2^)	23.4	23.2	22.3	24.1	23.4	24.7	23.4	23.4	**23.5 ± 0.5**
Body fat (%)	9.5	10.8	9.7	11.1	9.2	10.4	9.8	10.6	**10.1 ± 0.7**
VO_2peak _(mL·kg^-1^·min^-1^)	70.2	71.9	62.5	53.1	69.1	56.4	74.7	69.2	**66.4 ± 6.8**
HR_max _(bpm)	184	165	177	165	178	174	176	176	**174 ± 9**
VT (% HR_max_)	72	74	75	83	74	77	80	85	**77 ± 5**
RCP (% HR_max_)	91	89	90	89	91	89	90	92	**90 ± 1**
W_peak _(W·kg^-1^)	6.1	6.2	6.3	5.7	6.4	6.0	5.5	5.9	**6.0 ± 0.3**

BMI: body mass index; VO_2peak_: peak of oxygen uptake; HR_max_: maximum heart rate; VT: ventilatory threshold expressed as % of HR_max_; RCP: respiratory compensation point expressed as % of the maximum heart rate; W_peak_: peak of power.

### Preliminary testing

One week prior to the competition, all our athletes reported to a physiology laboratory to perform an incremental VO_2_max test under controlled conditions (22 ± 1°C, 40 - 60% relative humidity, 760 - 770 mmHg barometric pressure). They were asked to refrain from caffeine, alcohol and heavy exercise on the day before the tests, and to report to the laboratory at least two hours after having eaten. An incremental test was performed on an electronically braked cycle ergometer (Excalibur Sport, Lode, The Netherlands) modified with clip-on pedals. The exercise protocol started at 25 watts (W) and was increased by 25 W every minute until voluntary exhaustion. The pedaling cadence was individually chosen within the range of 70 - 100 revolutions per minute (rpm). During the test, oxygen uptake (VO_2_), minute ventilation (V_E_), carbon dioxide production (VCO_2_) and respiratory exchange ratio (RER) were measured, breath-by-breath, using a computerized gas analyzer (Cosmed Quark PFT-Ergo, Italy). Before each test, the ambient conditions were measured and the gas analyzers and inspiratory flowmeter were calibrated using high-precision calibration gases (16.00 ± 0.01% O_2 _and 5.00 ± 0.01% CO_2_, Scott Medical Products, USA). Respiratory data were averaged at 30 s intervals to determine VO_2_max taken as the highest average value. The ventilatory threshold (VT) and the respiratory compensation point (RCP) were measured by three independent reviewers according to methods described by Wasserman et al. [21]. In addition, heart rate was continuously recorded using a portable heart rate monitor (Polar RS800 SD, Finland). Heart rate data were averaged at 10 s intervals and the maximum heart rate was defined as the heart rate achieved at the point of exhaustion.

### Nutritional data

After the test all the athletes received nutritional guidelines and were encouraged to follow a high carbohydrate diet during the three days prior to the competition in order to optimize their glycogen replenishment. However, during the competition, there were no constraints and the nutritional pattern was programmed by the cyclists themselves. Furthermore, they received no direct instructions from the investigators during the event. Seven trained investigators were divided among the boxes weighing and recording all the food and fluid ingested by each participant during the recovery periods. To weigh all the food, we used two digital scales (Soehnle 8020, Spain) with a precision of 1 g increments up to 1 kg and 2 g between 1 and 2 kg. During the race, it was forbidden to provide to the athletes food and fluids in any point of the circuit with the exception of the box. All the food and fluids that cyclists consumed before every relay were weighed and recorded by the researchers. Immediately after every relay, food and fluids were weighed and recorded by the researchers again. The difference in weight was considered as the amount of food and fluids ingested by the cyclists during exercise. The type of food and fluids of sport products such as energy bars and gels ingested by the cyclists were described and recorded using the labels of the products. Information derived from prepared foods such as pasta, rice or sandwich was provided asking the form of preparation, directly, to the cyclists. The nutritional data was analyzed for nutrient composition using nutritional software. To guarantee a more accurate conversion of energy and nutrient intakes, we used a database of food from the country where the study was carried out (CESNID 1.0, Barcelona University, Spain). Information about the nutritional content of food not available in the computer program was obtained from the manufacturer. We divided the ingestion of energy derived from solid and fluid food (*i.e*. classified as products that did not need mastication).

Each subject was weighed 30 minutes prior to the race, after every cycle session and immediately after finishing the competition. The subjects were always weighed in clothing, shoes and bicycle helmets in order to facilitate the collection of the research data during the event. Weights were measured on calibrated scales placed on a hard level surface.

### Load of exercise and energy expenditure

During the whole competition, the heart rate was continuously monitored beat-by-beat using portable heart rate monitors (Polar RS800 SD, Finland). Later, all heart rate data were averaged at 10 s intervals. In order to establish a reference for heart rate, we identified three zones of physical exertion based on the VT and the RCP: zone I, below to the VT; zone II, between VT and RCP; and zone III, above RCP. In addition, to estimate the total work load of exercise performed by subjects we used the training impulse (TRIMP) method by Foster et al. [22]. To calculate TRIMP, the score for each heart rate zone was computed by multiplying the accumulated duration in this zone by a multiplier for this particular phase, e.g. 1 min in zone I was given score of 1 TRIMP (1 × 1), 1 min in zone II was given a score of 2 TRIMP (1 × 2), and 1 min in zone III was given a score of 3 TRIMP (1 × 3). The total TRIMP score was obtained by summating the results of the three zones [(min of zone I HR [< VT] × 1) + (min of zone II HR [> VT - < RCP] × 2) + (min of zone III HR [> RCP] × 3)].

To estimate energy expenditure during the race, the individually derived linear relationship between heart rate and VO_2 _was used to estimate the oxygen cost during the work efforts (r^2 ^= 0.988 ± 0.005). Two different individualized equations were established: 1) a linear regression equation for racing time which was derived from data during the incremental exercise test. We used an energy equivalent of oxygen based on the mean intensity during racing time (*i.e*. the non-protein energy equivalent corresponding to mean heart rate during the work efforts). This value was, on average, 0.02 MJ/LO_2 _(4.970 ± 0.048 kcal/LO_2_), corresponding to a RER of 0.941 ± 0.057 [23]. 2) A single exponential equation best fitted to VO_2 _and heart rate was taken during the recovery period of the cycle ergometer test (r^2 ^= 0.912 ± 0.015). An energy equivalent of 0.02 MJ/LO_2 _(4.825 kcal/LO_2_) was used, assuming a RER of 0.82 [23]. The rationale for our approach was that athletes performed bouts of exercise in which the heart rate-VO_2 _relationship can be assumed to be linear, interspersed with periods of recovery and rest, during which the heart rate-VO_2 _relationship becomes nonlinear [24].

### Statistical analyses

Data are presented as individual values and means ± SD. A non-parametric Wilcoxon test was used to compare the energy balance and changes in body mass and exercise intensity during the event. In addition, differences between nutritional data during the first (1900 h - 0700 h) and the second (0700 h - 1900 h) 12 hour period were assessed. The main nutritional variables (*i.e*. energy, carbohydrates, proteins, fats, fluid, sodium and caffeine) were correlated to speed and distance completed in absolute (*i.e*. km; km/h) and relative (*i.e*. % of decrease of distance and speed) values using Spearman's rank correlation analysis. In addition, fluid and sodium consumption was related to body weight loss. Non-parametric methods were applied, as not all parameters were ideally normally distributed. For all statistical tests, the significance level was set to *P *< 0.05. Data were analyzed using SPSS for Windows, version 15.0 (SPSS, Inc, Chicago, Ill).

## Results

### Performance during the event

**Figure 1:**
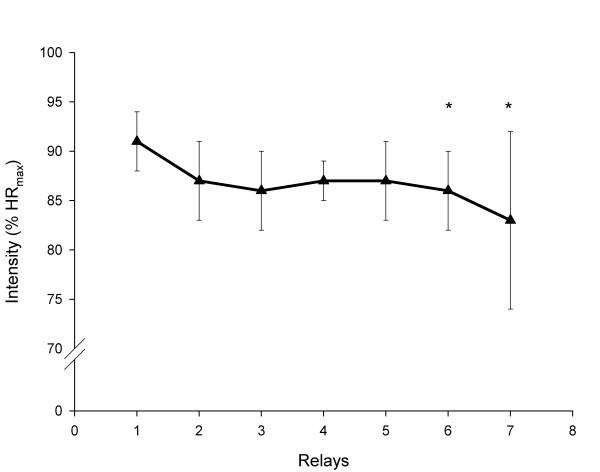
**Evolution of the intensity, expressed as % of maximum heart rate (HR_max_), during the event**. * Statistical difference (*P *< 0.05) mean intensity between the first relay compared with the sixth and seventh relay.

The main variables controlled during the race are summarized in Table 2. All participants finished the race although two athletes (number 4 and 8 on the Tables 1 to 4) reported gastro-intestinal disturbances during the last hours. All cyclists completed six work efforts, except for two riders who completed seven (subjects number 2 and 5 on the Tables 2 to 5). The mean intensity decreased significantly in riders performing six work efforts (1^st ^work effort: 91 ± 3% of maximum heart rate [HRmax]; 6^th ^work effort: 86 ± 4% of HRmax; *P *= 0.004) and also those completing seven (1^st ^work effort: 90 ± 5% of HRmax; 7^th^work effort: 83 ± 9% of HRmax; *P *= 0.002) (Figure 1). The mean cumulative climb during the race was 3168 ± 636 m. The cyclists rested between bouts of exercise for 173.2 ± 15.6 min.

**Table 2:** Performance during the event.

Subjects	1	2	3	4	5	6	7	8	Mean ± SD
Racing time (min)	358	406	381	303	495	330	299	318	**361 ± 66**
Average intensity (% HRmax)^a^	88.4	85.3	83.7	90.8	82.4	88.1	87.5	89.8	**87.0 ± 2.9**
Time spent in zone I (min)^b^	39	30	63	7	81	56	34	78	**49 ± 26**
Time spent in zone II (min)^b^	207	223	225	89	345	111	140	121	**183 ± 84**
Time spent in zone III (min)^b^	112	153	93	207	59	163	129	119	**129 ± 45**
TRIMP	789	935	792	806	948	767	697	677	**801 ± 98**
Distance (km)	207	223	208	165	282	182	171	163	**200 ± 40**
Average speed (km/h)	34.7	33.0	32.8	32.7	34.9	33.1	33.9	30.8	**33.2 ± 1.3**
Recovery time (min)	1082	1034	1059	1137	945	1110	1141	1122	**1079 ± 66**

^a^: percentage of maximum heart rate; ^b^: time spent in each zone of exercise intensity during the racing time (zone I: below to the ventilatory threshold; zone II; between the ventilatory threshold and respiratory compensation point; zone III: above to the respiratory compensation point); TRIMP: training impulse.

### Macronutrient intake

Food and fluids rich in carbohydrates were the main source of energy consumed during the event (Table 3). The athletes consumed 395 ± 193 (5.4 ± 2.6 g/kg; 42 ± 10%, respectively) and 549 ± 141 g of carbohydrates (7.7 ± 2.1 g/kg body mass; 58 ± 10%, respectively) during the first (1900 - 0700 h) and the second (0700 - 1900 h) period, respectively. Carbohydrates reported as fluids and solids were 533 ± 175 g (56.8 ± 10.6%) and 410 ± 174 g (43.2 ± 10.6%), respectively. Protein intake was heterogeneous, while three athletes ingested at rates above 2.5 g/kg body mass; the intake of the remaining subjects were below 2.0 g/kg body mass. Relative to time, the athletes ingested 92 ± 90 g (1.3 ± 1.2 g/kg body mass; 53 ± 14%, respectively) of protein during the first half of the event and 82 ± 52 g (1.1 ± 0.7 g/kg body mass; 47 ± 14%, respectively) during the second. The intake of lipids also differed; four riders showed intake rates below 1.0 g/kg and the other four between 1.6 g/kg and 2.7 g/kg, respectively. During the first 12-hour period, the cyclists ingested 59 ± 35 g (0.8 ± 0.5 g/kg of body mass; 55 ± 13%, respectively) of lipid and 48 ± 31 g (0.7 ± 0.4 g/kg of body mass; 45 ± 13%, respectively) during the second period.

**Table 3:** Macronutrient intake during the event.

Subjects	1	2	3	4	5	6	7	8	Mean ± SD
**Carbohydrates**									
Solids (g)	284	392	290	252	695	323	668	378	**533 ± 175**
Fluids (g)	538	533	442	566	889	574	456	268	**410 ± 174**
Total (g)	822	925	732	818	1584	897	1124	647	**943 ± 295**
g/kg^a^	12.4	13.5	9.4	12.3	21.4	11.9	15.2	8.8	**13.1 ± 4.0**
g/min^b^	2.06	2.55	1.91	2.70	3.12	2.66	3.77	2.11	**2.61 ± 0.62**
%^c^	77.3	80.2	76.6	81.4	62.6	64.8	67.0	59.3	**71.1 ± 8.7**
**Protein**									
Solids (g)	62	66	41	37	262	146	126	128	**109 ± 75**
Fluids (g)	35	25	35	33	245	80	60	8	**65 ± 76**
Total (g)	97	91	76	69	507	226	186	136	**174 ± 146**
g/kg	1.5	1.3	1.0	1.0	6.9	3.0	2.5	1.9	**2.4 ± 1.9**
%	9.2	8.0	8.1	6.9	20.2	16.1	11.2	12.7	**11.5 ± 4.6**
Ratio CHO: P (g)^d^	8.5	10.2	9.6	11.9	3.1	4.0	6.0	4.7	**7.2 ± 3.2**
**Lipids**									
Solids (g)	47	52	48	37	159	91	142	131	**88 ± 49**
Fluids (g)	17	10	17	15	42	25	22	5	**19 ± 11**
Total (g)	64	62	64	52	201	116	164	136	**107 ± 56**
g/kg	1.0	0.9	0.8	0.8	2.7	1.6	2.3	1.9	**1.5 ± 0.7**
%	13.7	12.2	15.3	11.8	18.0	18.6	22.2	28.3	**17.4 ± 5.6**

^a ^Ratio between total macronutrient intake (g) and body mass (kg) at the beginning of the event.

^b ^Ratio between total carbohydrate intake (g) and total racing time (min)

^c ^Percentage of the total energy intake

^d ^Ratio CHO: P (g): Ratio of total grams of carbohydrate intake in relation to total grams of protein during the event.

### Fluid, sodium and caffeine intake

**Figure 2:**
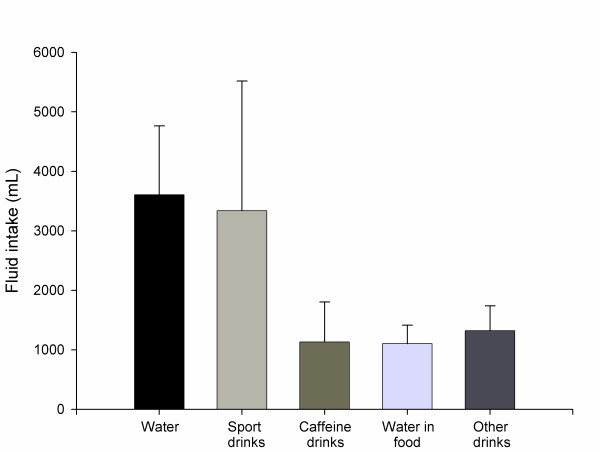
**Main fluids used for hydration and their average consumption during the event**.

Total fluid balance and sodium intake are illustrated in Table 4. Overall fluid consumption during the first half of the race (1900 - 0700 h) was 4794 ± 1633 mL (46 ± 7%) and 5703 ± 1421 mL (54 ± 7%) during the second (0700 - 1900 h), respectively. In relation to racing and recovery time, the cyclists ingested 907 ± 90 and 285 ± 128 mL/h, respectively. Overall fluid consumption showed that water (150 ± 48 mL/h) and sports drinks (139 ± 91 mL/h) were the main fluids ingested (Figure 2). The average sodium intake was 1189 ± 929 mg (5.2 ± 2.6 mmol/L of total fluid intake) and 3144 ± 2128 mg (17.8 ± 10.2 mmol/L) in fluids and solids, respectively. Consumption of sodium increased significantly (*P *< 0.05) during the second period of the competition (3083 ± 2020 mg; 71 ± 12%, respectively) compared to the first (1250 ± 898 mg; 29 ± 12%, respectively). Body mass decreased (3.0 ± 1.3%) significantly over the event (pre-race: 72.0 ± 4.4 kg; post-race: 69.9 ± 4.9 kg; *P *= 0.012). The loss of body weight during the first (1900 - 0700 h) and the second half (0700 - 1900 h) of the event was 1.1 ± 0.9 kg and 1.9 ± 0.6% (*P *= 0.273), respectively. We found no statistical relationship between both fluid intake (*r *= 0.024; *P *= 0.943) and sodium intake (*r *= 0.095; *P *= 0.823) with body weight loss.

**Table 4:** Fluid, sodium and caffeine intake and body mass loss during the event.

Subjects	1	2	3	4	5	6	7	8	Mean ± SD
**Fluid intake**									
Racing time (mL/h)	923	821	854	888	911	841	1110	905	**907 ± 90**
Recovery time (mL/h)	291	352	94	283	522	316	261	163	**285 ± 128**
Total (mL)	11185	11293	7106	9850	15831	10535	10480	7699	**10497 ± 2654**
**Sodium**									
Fluids (mg)	911	897	518	767	3,321	1,682	678	738	**1189 ± 929**
Solids (mg)	2466	2240	981	1583	6424	1357	4027	6073	**3144 ± 2128**
Total (mg)	3377	3137	1499	2350	9745	3039	4705	6811	**4333 ± 2714**
**Body mass loss (kg)**	2.8	1.4	1.3	2.5	2.3	3.0	0.8	3.2	**3.0 ± 1.3**
**Caffeine (mg/kg)**	2.0	2.7	2.4	1.2	3.4	0.1	2.5	1.5	**2.0 ± 1.0**

The total consumption of caffeine was 142 ± 76 mg (2.0 ± 1.0 mg/kg body mass) (Table 4). The consumption of caffeine increased significantly (*P *< 0.05) during the last 12 hour period of the event (99 ± 50 mg; 1.4 ± 0.7 mg/kg body mass) compared with the first 12 hours (43.9 ± 49.5 mg; 0.6 ± 0.7 mg/kg body mass). Caffeinated beverages were the main caffeine containing fluids ingested, and smaller amounts of caffeinated drinks, such as Red Bull^®^, coffee, and carbohydrate gels with added caffeine, were ingested by some athletes (Figure 2).

### Energy balance

The individual and mean values of energy intake are summarized in Table 5. Energy intake (22.8 ± 8.9 MJ) was significantly lower than energy expenditure (42.9 ± 6.8 MJ; *P *= 0.012). Thus, a high proportion of energy (54 ± 19%) expended by the athletes was provided from the endogenous fuel stores (Table 5). During the first 12-hour period (1900 - 0700 h), the athletes consumed 10.8 ± 5.6 MJ (47 ± 7%) and 12.0 ± 3.6 MJ (53 ± 7%) during the second period (0700 - 1900 h), respectively. Solid foods were the main source of ingested energy reported as 52 ± 12% of the total energy intake. The remaining 48 ± 12% of ingested energy was supplied by fluids. Energy intake while racing was lower (3.7 ± 1.1 MJ; 16 ± 5%) and derived only from fluids such as hypotonic beverages and gels. The cyclists used mainly the resting periods to ingest food and beverages (19.1 ± 7.0 MJ; 84 ± 5%).

**Table 5:** Energy balance during the event.

Subjects	1	2	3	4	5	6	7	8	Mean ± SD
**EI during racing time (MJ)**^a^									
Fluids	2.5	3.1	3.1	2.6	5.9	4.7	3.7	3.9	**3.7 ± 1.1**
**EI during recovery time (MJ)**									
Solids	7.6	9.6	7.6	6.2	22.0	11.3	18.7	13.4	**12.1 ± 5.7**
Fluids	7.7	6.6	5.4	8.0	14.7	7.1	5.7	0.9	**7.0 ± 3.8**
Total Energy Intake	17.8	19.3	16.1	16.8	42.6	23.1	28.1	18.2	**22.8 ± 8.9**
**Energy expenditure (MJ)**									
Racing time	32.6	30.1	34.3	22.1	40.1	25.5	22.5	22.8	**28.8 ± 6.6**
Recovery time	13.4	13.9	20.0	13.0	9.4	14.2	14.5	15.6	**14.3 ± 2.9**
Total Energy Expenditure	46.0	44.0	54.3	35.1	49.5	39.7	36.0	38.4	42.9 ± 6.8*
**Energy Deficit (MJ)**	-28.2	-24.7	-38.2	-18.3	-6.9	-16.6	-7.9	-20.2	-**20.1 ± 10.4**
**EI:EE**^b^	0.39	0.44	0.30	0.48	0.86	0.58	0.76	0.47	**0.54 ± 0.19**

^a ^Energy intake

^b ^Ratio between energy intake and energy expenditure.

* Statistical difference (*P *< 0.05) between total energy intake and energy expenditure during the event.

### Correlation between nutritional data and performance during the event

The main performance variables such as distance covered and speed did not correlate to the main nutritional variables such as calories, carbohydrates, fluids and caffeine (*P *< 0.05). In addition, other dietary variables such as intake of proteins, fats and sodium were also not related to performance variables. The strongest correlation was found between cycling speed and total fluid intake (*r *= 0.71; *P *= 0.074). When we compared data between the first and the second half of the event, the strongest correlations were found between the total fluid intake in mL/h (r = -0.66; *P *= 0.073) and mL of racing time (r = -0.66; *P *= 0.077) with % of speed decrease during the last 12 hours (0700 - 1900 h).

## Discussion

In contrast to our first hypothesis, this study shows that athletes were able to consume amounts of carbohydrates which were in accordance with the current recommendations for longer events [6,7]. However, despite of this fact, these athletes did not meet their energy requirements during the event resulting in a higher energy deficit. The huge workload performed by athletes (TRIMP > 800), which was significantly above to data reported in elite cyclists during high mountain stages of the Tour de France (~ 600 TRIMP) [25], induced a higher energy expenditure. Thus, these results confirmed partially our preliminary hypotheses and were in agreement with two previous investigations showing that, like solo events, a high energy deficit is common in a team relay format events despite that athletes have considerable time to recover between bouts of exercise [4,26]. One explanation for this effect has been related with appetite suppression since it is known that longer exercise induces a suppression of acylated ghrelin in humans [27]. Ghrelin is an amino acid peptide hormone secreted primarily from cells within the stomach and it has been suggested to have an orexigenic function (*i.e*. appetite stimulating) [27].

### Macronutrients intake

The recommended amount of carbohydrate intake during longer exercise to optimize oxidation rates have been reported as between 1.0 to 1.5 g/min [15]. This recommendation could be also useful to improve glycogen replenishment during the first 4 hours after exercise [28]. In the current study, the mean carbohydrate intake in relation to total racing time (2.61 ± 0.62 g/min) was substantially above these values. Moreover, the relative amount of carbohydrate intake by cyclists was equivalent to 13.1 ± 4.0 g/kg of body mass. This data reflects the recommendations for extremely prolonged and intense exercise (10-12 g/kg of body mass/day) [11]. These findings show that ultra-endurance athletes competing in team relay format can reach the consumption of carbohydrates which has been suggested in a laboratory study to optimize carbohydrate oxidation [13]. This fact is very important in ultra-endurance team relay events, since athletes can perform more than 80% of racing time at intensities corresponding to zone II and III of HR_max _(Table 2). It is known that this pattern of exercise elicits an important oxidation of carbohydrates as a main fuel for muscle contraction [12].

Nevertheless, not only is the amount of carbohydrates important, it should be also paid attention on other factors relating to the limitations of carbohydrate absorption. The feeding schedule, particle size, meal temperature, osmolality and exercise intensity determine the gastric emptying and absorption in the duodenum [29]. For instance, some studies have demonstrated that a homogenized fluid meal, rich in carbohydrates, empties substantially faster than an equivalent solid meal [29,30]. However, in longer events, solid food will satisfy an athlete's hunger and allow for more variation, which can also help to intake adequate amounts of carbohydrates [1]. In this study the source of energy was balanced between solids (2,877 ± 1,355 kcal) and fluids (2,560 ± 1,074), respectively.

In addition, there is evidence that during high-intensity exercise (> 80% VO_2_max) a reduced blood flow to the gut may result in a decreased absorption of both glucose and water [31]. In the current study, two cyclists evidenced gastro-intestinal disturbances related to nausea, abdominal cramps and diarrhea during the last hours of the event. Interestingly, both cyclists performed relays at high intensity compared with the other cyclists (subject's number 4 and 8 in Table 2). Taking in account that blood flow to the gut decreases in proportion to the exercise intensity and gastro-intestinal problems are more likely to occur when the exercise intensity is increased [23], this fact could be an explanation for the occurrence of these problems. However, this is only speculation and we cannot exclude other important factors that may also increase the risk of gastro-intestinal disturbances. For instance, an interesting finding of this study was that fluid yogurt represented the third highest energy contribution in the diet of the cyclists (Table 6). Although the ingestion of milk and derived products just after exercise has been suggested to be an excellent dietary form to attenuate whole body protein breakdown [32], there is also evidence indicating that the consumption of such products could be associated with greater satiety and reduced *ad libitum *energy intake in humans [33]. It seems that this effect is related with the presence of casein proteins in milk [34]. Thus, although the consumption of milk or derived products like liquid yogurt could be an easy form to enhance recovery processes after bouts of exercise during team relay event, it should be taking in account that they can decrease gastric emptying and be a risk for intestinal disorders. To avoid these problems, we recommend that athletes need to practice their dietary strategy before the event testing the tolerance of all products that they will use during the race. In addition, like muscle skeletal adaptations induce by physical training, adequate nutritional training -ingestion of small and frequent amounts of food and fluids during exercise- may induce adaptations of the digestive system and reduce the risk of gastro-intestinal distress [31].

**Table 6:** Main food and beverages sources of energy and nutrients during the event.

Food	Energy contribution (%)
Pasta and rice (with tomato or oil olive and cheese)	25.0
Sport drinks	13.8
Fluid yogurt	12.3
Caffeinated drinks (Cola and Red Bull)	8.5
Fruits (Banana, apple, peach and pear)	5.6
Cakes	5.1
Meat (Chicken and ham)	4.6
Sport Bars	4.1
Sport Gels	3.6
Bread	3.3
Fruit juice	2.9
Dried fruits (almonds and nuts)	2.2
Cereals	2.0
Milk	1.9
Tuna	0.4
Others (protein supplements, coffee, soy milk, sugar, etc)	4.7

Regarding protein recommendations (1.2 to 1.7 g/kg of body mass/day) [11], we found that almost all athletes consumed an adequate amount of this macronutrient. However, although protein is not an essential substrate used to provide energy, it could play an important role during longer events. Several studies have suggested that a carbohydrate/protein ratio around 4:1 can enhance glycogen recovery, as well as protein balance, tissue repair and adaptations involving synthesis of new protein [35,36]. These findings are interesting for ultra-endurance athletes competing in team relay events because the nutritional goal of them is to promote and accelerate the recovery of their endogenous glycogen stores and fluid replenishment after every work effort. However, the ingestion of carbohydrate/protein ratio of 4:1 in competition like the current event induces higher protein consumption. For example, applying this ratio to this study, it was estimated that adequate protein consumption would have to be ~ 236 g (~ 3.6 g/kg body mass). In the present study, only two cyclists were able to consume amounts of protein like this. Furthermore, apart of these supposed benefits of carbohydrate and protein combination, it should be also taken in account that protein intake is associated with greater satiety and a reduced *ad libitum *energy intake in humans [33]. Therefore, further studies are needed to analyze whether an increase of protein intake above the current recommendations (1.2 to 1.7 g/kg of body mass/day) may induce benefits in longer and high-intensity sport events.

Lastly, fat intake in these athletes was low in comparison with previous studies involving also cyclists during team relay events [26]. This fact, clearly, contributed to increase the negative energy balance showed in this study. However, contrary to carbohydrates, there is no evidence indicating that the increase of fat intake improves exercise performance [37]. The stores of fat in the human body are so large and they will not become depleted after prolonged events such as 24-hour competitions [38]. Thus, there is no evidence to justify that the current cyclists would increase the amount of fat intake during the event. Nevertheless, the inclusion of fat in the diet of ultra-endurance events could be interesting, not to provide caloric dense options, but to satisfy the taste of foods [1].

### Fluid balance and caffeine intake

The volume of fluid ingestion during bouts of exercise was in accordance with the recommendations for longer events [16]. However, the composition of fluids was not in accordance with these guidelines [16]. While these riders ingested high amounts of water, they should have prioritized the consumption of hypotonic fluids containing carbohydrates, such as sucrose, maltose or maltodextrin at ~3-8% weight/volume, and sodium concentration of between 30 and 50 mmol/L [39]. The consumption of these beverages is interesting in order to reduce dehydration and weight losses. In this study, the body mass of the riders decreased significantly after the race being this reduction more important in the second half of the event compared with the first 12 hours. However, it is worth to mention that all body mass reduction cannot be related to fluid losses, since we found no relationship between body weight losses and fluid ingestion. From this viewpoint, there is evidence that other factors such as loss of fat mass, skeletal muscle mass, glycogen and water stored in glycogen could also account for at least 2 kg of body mass loss [40,41]. Thus, and according to the high energy deficit in the present cyclists, it could be also suggested that a considerable amount of body weight loss was derived from losses of their endogenous energy stores. Unfortunately, we did not record urine output during the study. These data might have provided more detailed information about fluid balance and the origin of body weight loss. In addition, the use of sweat patches could be very interesting to analyze electrolyte losses in future investigations.

Products rich in caffeine such as caffeinated beverages, coffee and caffeinated sport gels were consumed especially during the second half of the event when fatigue symptoms were more pronounced. Doses of caffeine between 1.5 and 3.5 mg/kg^-1 ^body mass have been reported to enhance power output in laboratory studies [18]. Although, caffeine has been also linked to diuretic effects [42], it seems that moderate doses (< 460 mg) of caffeine, do not induce water and electrolyte imbalance or hyperthermia [42]. In this study, all the subjects consumed amounts of caffeine below this threshold during the event.

### Relationship between nutritional data and performance during the event

In the present study, we found no correlation between the main nutritional variables (*i.e*. energy, carbohydrates, fluids and caffeine) and performance (*i.e*. completed distance or mean cycling speed) during the event. The strongest relationship was found between total fluid intakes and cycling speed. This fact can add support to the wide scientific evidence indicating that in hot environmental conditions, such as in the current event, a careful hydration strategy is one of the key fundamentals to maintain the athletic performance [16,39].

### Strength and limitations of the present study

A major strength of this study is the careful nutritional analysis which was carried out in a community and setting where little information has been forthcoming. We were able to weigh and record all foods and fluids ingested by the eight athletes in a real competition. This methodology is not easy to apply in the field, but reports more reliable information compared to questionnaires or dietary surveys which have been employed in other previous investigations [9,10,43,44]. However, we should also acknowledge some limitations and caveats in this study. Perhaps, the main limitation was the sample size, which was small to analyze the relationship between nutritional and performance variables. In addition, although the relationship between heart rate-VO_2 _has been shown to be an acceptable measure for estimating energy expenditure during non-steady state [45,46], it should be admitted that this methodology can be affected by several physiological and environmental factors such as dehydration and temperature [47]. Currently, doubly-labeled water is considered to be the gold standard method for estimating energy expenditure in free living humans, which can also be used under field conditions, but it is an expensive method. On the contrary, the heart rate-VO_2 _regression equation is a feasible and reasonably priced method which has been employed in other previous investigations [43,48,49].

## Conclusions

Cycling ultra-endurance events lasting 24-hour in a team relay format elicits several bouts of exercise, with limited recovery between them, at high exercise intensity (> 75% of VO_2_max). This pattern of exercise stimulates an important consumption of carbohydrates to supply energy for muscle contraction. This study shows that these ultra-endurance athletes were able to consume large amount of carbohydrates in a field competition which was in accordance with data obtained in laboratory studies in order to optimize carbohydrate oxidation during exercise. However, despite of this fact we found an increased energy deficit throughout the race. This finding indicates that the nutritional pattern followed the days before to the competition could be even, or at least, as important that the dietary strategy during the event. In addition, although protein ingestion by athletes was in accordance with the current recommendations for athletes, it has been suggested that carbohydrate/protein ratio of 4:1 can enhance several metabolic processes such as glycogen recovery, protein balance and tissue repair which can affect the overall performance. Nevertheless, while the accomplishment of this ratio could induce these benefits and reduce the energy deficit it is unknown whether athletes can tolerate high protein ingestion and perform high intensity exercise without gastro-intestinal disturbances. To avoid these problems, it is very recommendable that athletes perform a nutritional training before to the event ingesting small and frequent amounts of macronutrients and fluids during training sessions. This training may enhance the response of the digestive system during ultra-endurance events and reduce the risk of gastro-intestinal distress during longer events in hard environment conditions.

## Competing interests

The authors declare that they have no competing interests.

## Authors' contributions

RB, participated in the design of the study, managed the data collection process, conducted the analysis and drafted the manuscript. FR and XI, participated in the design of the study and managed the data collection process. AB, MM, JP, PT and JV participated in the data collection process. BK and TR supervised the analyses of data and helped to draft the manuscript. All authors read and approved the final manuscript.
